# HIV Viral Load Testing in the South African Public Health Setting in the Context of Evolving ART Guidelines and Advances in Technology, 2013–2022

**DOI:** 10.3390/diagnostics13172731

**Published:** 2023-08-22

**Authors:** Lucia Hans, Naseem Cassim, Somayya Sarang, Diana Hardie, Silence Ndlovu, W.D. Francois Venter, Pedro Da Silva, Wendy Stevens

**Affiliations:** 1 Department of Molecular Medicine and Haematology, Faculty of Health Sciences, University of Witwatersrand, Johannesburg 2193, South Africa; 2 Wits Diagnostics Innovation Hub, Faculty of Health Sciences, University of Witwatersrand, Johannesburg 2193, South Africawendy.stevens@wits.ac.za (W.S.); 3 National Health Laboratory Service, National Priority Programme (NPP), Johannesburg 2193, South Africa; 4 National Health Laboratory Service, Cape Town 8005, South Africa; 5 Division of Medical Virology, Faculty of Health Sciences, University of Cape Town, Cape Town 7700, South Africa; 6 Ezintsha, Faculty of Health Sciences, University of the Witwatersrand, Johannesburg 2193, South Africa; fventer@ezintsha.org

**Keywords:** HIV viral load, national program, guidelines, advances, technology, South Africa

## Abstract

HIV viral load (VL) testing plays a key role in the clinical management of HIV as a marker of adherence and antiretroviral efficacy. To date, national and international antiretroviral treatment recommendations have evolved to endorse routine VL testing. South Africa (SA) has recommended routine VL testing since 2004. Progressively, the centralised HIV VL program managed by its National Health Laboratory Service (NHLS) has undergone expansive growth. Retrospective de-identified VL data from 2013 to 2022 were evaluated to review program performance. Test volumes increased from 1,961,720 performed in 2013 to 45,334,864 in 2022. The median total in-laboratory turnaround time (TAT) ranged from 94 h (2015) to 51 h (2022). Implementation of two new assays improved median TATs in all laboratories. Samples of VL greater than 1000 copies/mL declined steadily. Despite initial increases, samples of fewer than 50 copies/mL stagnated at about 70% from 2019 and declined to 68% in 2022. Some variations between assays were observed. Overall, the SA VL program is successful. The scale of the VL program, the largest of its kind in the world by some margin, provides lessons for future public health programs dependent on laboratories for patient outcome and program performance monitoring.

## 1. Introduction

South Africa (SA) is home to the largest population of people living with HIV (PLWHIV) globally, reported to be 8.45 million in 2022 [1]. Accordingly, the SA National Department of Health (NDoH) operates the world’s largest antiretroviral (ARV) program, with an estimated 5.5 million people receiving treatment [2]. Antiretroviral treatment (ART) programs are increasingly relying on laboratory tests as an adjunct for optimal patient care. The HIV viral load (VL) is an essential tool in the clinical management of PLWHIV. As early as 1996, the VL was shown to be a surrogate measure of response to ARTs [3] and a sustained response predictive of improved morbidity and mortality outcomes [4,5] Additionally, VL thresholds identify patients who are virologically suppressed (VS) or failing ART (VF); more frequent tests are recommended as part of the management of non-suppression and failure events, which may vary according to guidelines [6,7,8].

ARV guidelines directly influence laboratory testing practices. The World Health Organization (WHO) guidelines are adopted unchanged or adapted for national guidelines in resource-limited settings. These clinical recommendations steer laboratory testing algorithms, and consequently influence test request volumes and assay selection. A series of WHO recommendations saw general ART eligibility progress from a CD4 count <200 cells/µL and/or WHO clinical stage 4 in 2003, through <350 cells/µL and <500 cells/µL and WHO stage 3/4 in 2010 and 2013, respectively, as evidence for earlier therapy accumulated, and as antiretroviral therapy became safer [9,10,11]. All PLWHIV were eligible in 2016, irrespective of their CD4 count [8] VL monitoring is recommended at six-to-twelve-monthly intervals [12]. Currently, the WHO VF threshold of a confirmed VL greater than 1000 cp/mL on two consecutive occasions is unchanged from 2013, when it was reduced from the 5000 copies/mL threshold recommended in 2010. The VS. threshold of less than or equal to 50 copies/mL was introduced in 2021 [12]. 

Unusual in Africa at the time, VL was already routinely recommended at six-monthly intervals in the first version of the SA state ARV Guidelines that was published in 2004, with failure set at 5000 copies/mL [13]. Several updated recommendations in the subsequent versions had implications for the VL testing program. In 2010, pregnant women and patients co-infected with tuberculosis (TB) presenting with CD4 ≤ 350 cells/µL were eligible to initiate ARV, while the CD4 ≤ 200 µL remained in place for others and the diagnosis of VL failure now required two consecutive VLs of ≥1000 copies/mL three months apart [14]. The CD4 ≤ 350 cells µL was applicable to all by 2013 [15] and was raised to ≤500 cells/µL in 2014 [16]. Universal treatment was implemented in 2016 [17]. In 2019, suppression at <50 copies/mL was included, and VL > 50 copies/mL required the person to have a VL test at three months after enhanced adherence counselling [7].

The establishment of the Joint United Nations Programme on HIV/AIDS (UNAIDS) ‘90-90-90’ targets, focusing on 90% of people with HIV knowing their status, 90% of these being on treatment, and 90% in turn being virologically undetectable, and the launch of the ‘Undetectable = Untransmittable’ campaign underscored the need for the scaling up of testing services and the availability of accurate VL quantification at the clinically relevant thresholds [18,19,20,21]. Review of virological suppression rates at local, national and global levels monitors progress towards achievement of the third UNAIDS target [2]. The quality of the VL data reported is contingent on laboratory and comparative assay performance. 

VL assays have advanced beyond the less-sensitive, labour-intensive, and technologically complex commercial HIV-1 quantitative assays first approved by the Food and Drug Administration (FDA) [22,23]. Driven by clinical evidence that suppression improves clinical outcome early on in assay advancement, assay sensitivity quickly improved to a lower detection limit of less than 20 copies/mL [24]. Further developments produced enhanced specificity and precision in VL results over earlier assay versions. Current testing platforms are more sophisticated; enhancements include automation with primary tube sampling, increased test throughput, reduced time to test completion, lower sample input volumes, alternative sample types and analytical software upgrades permitting integration with laboratory information systems (LIS). 

The National Health Laboratory Service (NHLS) provides public sector laboratory services to more than 80% of the population in SA. HIV VL program operations are overseen by the National Priority Programme (NPP) of the NHLS which was established to execute the NDoH’s strategic health priorities by aligning laboratory and clinical activities related to priority tests [25]. VL testing is plasma-based and performed in centrally located laboratories using medium- and high-throughput molecular assays. Assays are selected by a national supply chain management (SCM) tender process, and the award implementation is tailored to meet programmatic and individual laboratory requirements. Since its inception, the VL program has executed three tender awards. The approximately six million plasma VL tests performed in 2022 reflect a mature program that has expanded appreciably since the first 34,000 tests reported in 2004 [26]. 

The study objectives were to describe the scale-up of molecular VL testing and to illustrate the impact of changing ARV guidelines and assay changes in levels of virological failure, suppression and turnaround-time performance. The SA HIV VL program provides a suitable setting for this evaluation.

## 2. Materials and Methods

A retrospective review of the NHLS HIV VL program was performed. Data from 2013 to 2022 were selected for the VL analysis. Although the VL program was operational before 2013, VL data for this period were not available for analysis, due to an LIS change. Secondary-level de-identified VL data containing the following variables were extracted from the NHLS corporate data warehouse: (i) year, (ii) laboratory, (iii) test volumes, (iv) VL result, (v) turnaround time and (vi) assay name. Extracted results were categorised into (i) <50 (virological suppression), (ii) 50–999 (low-level viremia) and (iii) ≥1000 (viraemia) copies/mL. For this analysis, clinical data were not available and, therefore, failure is reported based on a single result of ≥1000 copies/mL. Aggregate and assay-specific annual test volumes, median in-laboratory turnaround times (TAT), the percentage of samples reported within the expected 96 h total TAT target, VF, and VS. were determined. Total TAT is defined as time taken to produce a valid result and was measured in hours from sample registration in the referring laboratory until authorisation on the LIS. The time from sample collection to registration was not available, and the assay onboard time could not be reported because the assay start time was not interfaced with the LIS. Results for which assay names had not been transmitted were excluded from assay-specific TAT analyses. 

The dataset was prepared and analysed using Microsoft Excel (Microsoft Corporation, Redmond, WA, USA) and SAS 9.4 (SAS Institute, Cary, NC, USA). Choropleth maps were created using ArcGIS (ESRI, Redlands, WA, USA). 

## 3. Results

### 3.1. Laboratory Footprint and Assays in Use

**Figure 1:**
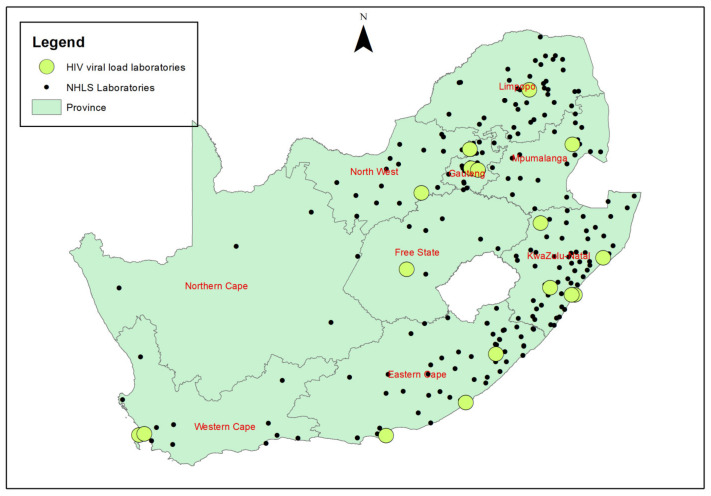
Map of National Health Laboratory Service (NHLS) sites in South Africa across nine provinces. Laboratories that offer HIV viral load testing are indicated.

From 2013 to 2015 there were 17 VL laboratories across eight of the nine provinces in SA. This was reduced to 16 in 2016/2015. following consolidation of testing at a larger laboratory in Kwazulu-Natal with a new tender award. An additional lab was added in Gauteng laboratory at the end of 2021 as part of a disaster recovery plan (Figure 1). 

Four assays manufactured by two suppliers were in use between 2013 and 2022. The COBAS^®^ AmpliPrep/COBAS^®^ TaqMan^®^ HIV-1 Test, version 2.0, V2 (CAPCTM) (Roche Diagnostics GmbH, Manheim, Germany) and the Abbott RealTime HIV-1 (m2000) (Abbott Molecular Inc, Des Plaines, IL, USA) assays were in already in use in 2013. New tenders were awarded in 2015 and 2019. The Roche cobas^®^ HIV-1 Quantitative nucleic acid test for use on the cobas^®^ 6800/8800 Systems (cobas) was introduced in 2015 and the Abbott Alinity m HIV-1 (Alinity) in 2019. At the end of 2021, the cobas assay was in use in seven laboratories and the Alinity in ten laboratories.

### 3.2. Test Volumes

A total of 45,334,864 tests were performed between 2013 and 2022 (Table 1). Total annual volumes tested increased from 1,961,720 in 2013 to 6,274,438 in 2022 (a 3.1-fold increase). Annual test volumes increased by 29%, on average, for the calendar years 2014 to 2016. The rate of increase decreased in the subsequent years and ranged from 17% in 2017 to 8.6% in 2019. In 2020 and 2021 the percentage increase declined to 2.1% and 1.4%, with recovery to 8.4% reported in 2022.

### 3.3. Turnaround Times 

**Figure 2:**
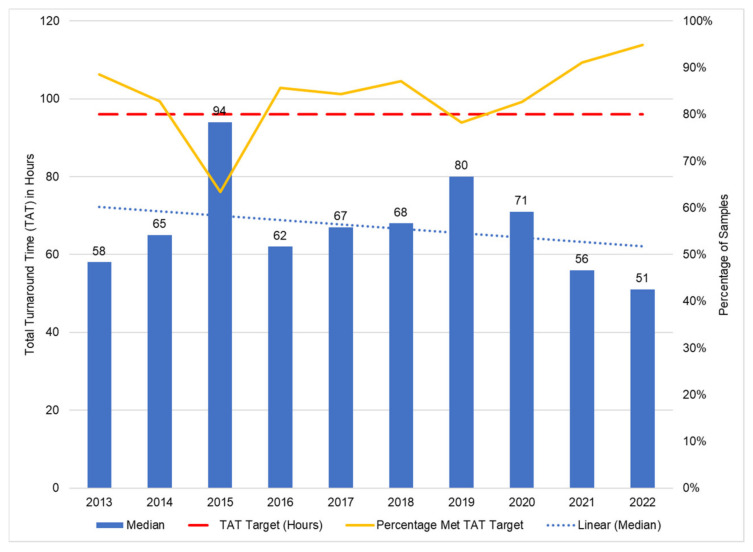
HIV VL TAT performance assessed using a target of 96 h for specimens received within the National Health Laboratory Service for the period between January March 2013 and December 2022. The median total TAT and percentage of specimens that met the TAT target are reported.

The median in-laboratory TAT ranged from 51 h in 2022 (IQR: 36–75) to 94 h in 2015 (IQR: 61–140). The percentage within the TAT target of 96 h improved from 63.5% in 2015 to 94.9% by 2022. The highest median TATs were reported during 2015 (cobas implementation, 94 h) and 2019 (Alinity implementation, 80 h), (Figure 2). The linear trend line indicates a downward trajectory for the median total TAT between 2013 and 2022.

The median in-laboratory TAT per platform ranged from 80 h (CAPCTM) to 50 h (Alinity). The percentage of samples that met the TAT target was 73.7% and 78.2% for the CAPCTM and m2000 assays, respectively (Table 2). This increased to 89.0% for the cobas compared to 91.7% for the Alinity assay. Laboratories that transitioned new assays reported a decreased median total TAT reduction of 19 (from m2000 to Alinity m) and 22 h (from CAP/CTM to 6800/8800), 7 h (m2000 to cobas) and 32 h (CAPCTM to Alinity) (data not shown).

### 3.4. Virological Failure and Suppression

**Figure 3:**
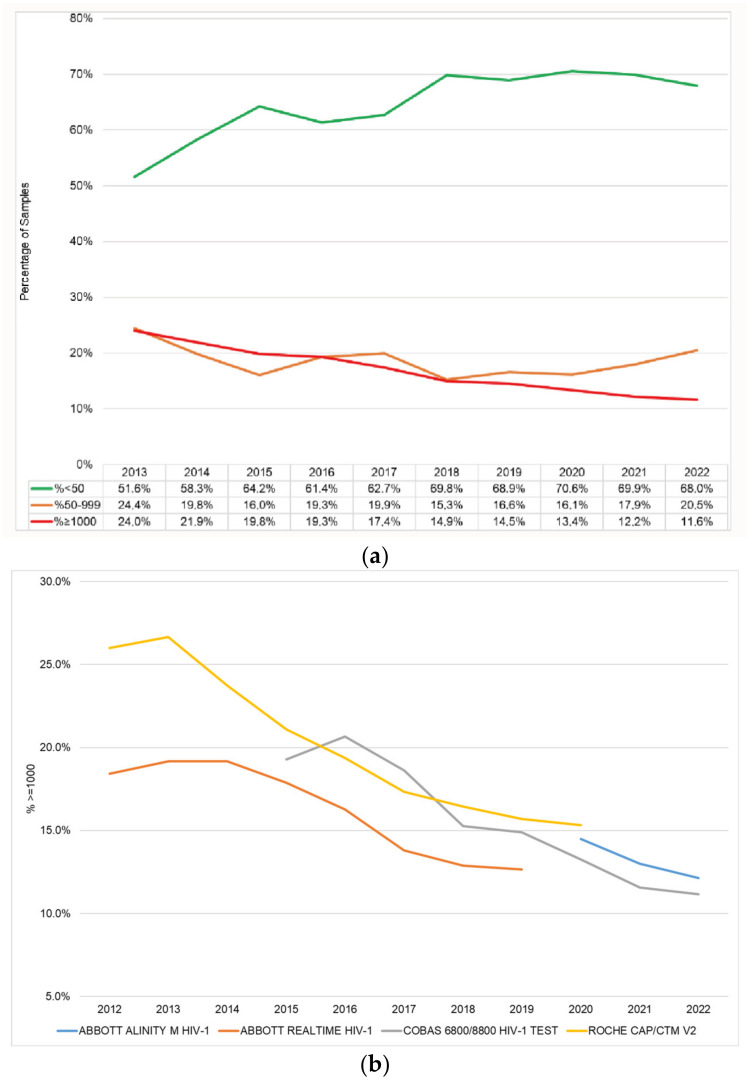

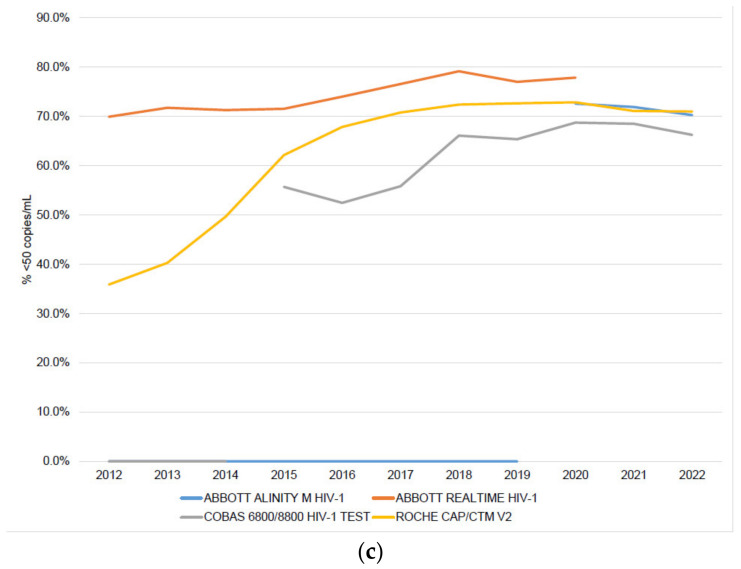
(**a**) Total percentages of samples for which VL results of <50, 50–999 and ≥1000 copies/mL were obtained from 2013 to 2019. (**b**) Percentages of samples reported as ≥1000 copies/mL per assay during period in use between 2013 and 2022. (**c**) Percentages of samples reported as <50 copies/mL per assay during period in use between 2013 and 2022.

The percentage of samples ≥1000 copies/mL decreased from 24.0% in 2013 to 11.6% by 2022 (Figure 3a). Failure rates remained steady during 2020 (13.4%) and 2021 (12.2%). The decrease in the percentage of samples ≥1000 copies/mL was observed across platforms (Figure 3b). The m2000 consistently reported lower percentages of samples ≥1000 copies/mL than the Roche assays. The cobas consistently reported a 1% lower percentage of samples of ≥1000 copies/mL compared to the Alinity.

Suppression rates mostly increased from 2013 to 2014, followed by a decline in 2015, recovered between 2016 and 2018, and then remained static at around 70%, from 2019 to 2021 (Figure 3a). A declining trend in <50 copies/mL and an associated increase in 50–999 copies/mL was observed in 2022. The m2000 consistently reported a higher percentage of samples of <50 copies/mL (Figure 3c). The Alinity reported 4% more samples of <50 copies/mL compared to the cobas, across time. 

## 4. Discussion

The NHLS HIV VL testing program recorded a three-fold increase in VL tests performed between 2013 and 2022. Rapid expansion was experienced during 2013 to 2017, followed by declines from 2017 to 2022. Here, VL data is unlinked to clinical history; each VL test does not represent an individual PLWHIV on ART, but rather a unique test request. Despite this caveat, the trend in volumes tested is similar to the trend predicted by Meyer-Rath et al. [27], who predicted a threefold increase in PLWHIV receiving ART for the period between 2010/2011 and 2016/2017, attributed to improved programmatic coverage and increased prevalence rather than increased eligibility. A decline in percentage increase started from 2016, a year later than predicted by Meyer-Rath, et al., and which, at 9% lower than 2015, did not meet the initial rapid rise forecast for the onset of universal treatment. The 2020 (2.1%) and 2021 (1.4%) could be attributed to the reduction in ARV initiations during the COVID-19 pandemic [28].

Median in-laboratory TAT varied across the years. The peak increases were observed during tender implementation periods. The percentage within the TAT target mirrored these trends, with decreases below 96% reported in 2015 and 2019. Instrument rollouts vary in complexity, depending on physical attributes of the laboratory site, and the physical requirements of the instrument, such as acceptable floor stability, sufficient air conditioner cooling capacity and appropriate power supply. The installation of the cobas 6800/8800 instruments in 2015 required renovations to most of the 13 new sites. Moreover, these instruments were relatively untried in the field, having been launched two years earlier. Engineers were trained, but inexperienced in the installation of these sophisticated platforms, resulting in significant downtime in laboratories during rollout. Anticipated teething problems like user-related errors due to new operators were realized, but the unanticipated errors related to real-world use in high-volume test settings, which impacted TAT (unpublished NHLS operations reports). Similarly, the rollout of the Alinity m instruments across ten sites in 2019 and 2020 had resulted in increased TAT, albeit with fewer delays than in 2015. Implementation was improved by utilizing lessons from the previous roll-out; staggering multiple installations in a single lab, ensuring that the new instrument was fully operational before decommissioning the older platform and ensuring that sample transport logistics were in place for smooth diversion to other laboratories, if required. 

Overall, median TAT and the percentage with a TAT target improved with the newer technologies, which was proven by the continuous downward trend observed from 2020 onwards. Although actual sample preparation and onboard test times were not compared, transition to these instruments appears to have improved laboratory workflow and time for reporting a valid result. The difference between the cobas and Alinity assay could be attributed to assay specifications, with the former batch-based with 94 samples and three assay controls required for a full run, and the latter permitting daily controls and smaller rack-based continuous sample loading procedures [29,30]. Specific manufacturer time-to-result claims also differed, with the Alinity producing a result in less than 115 min, compared to the approximately 3 h required for the cobas. Furthermore, cobas 8800 instruments were placed in the higher-volume laboratories, due to their higher throughput, suggesting that significant downtime would result in longer delays because of larger volumes of outstanding tests. Additionally, different laboratory operating hours, staff complements and workflows could all have contributed to the TAT differences noted, warranting more intensive research to interrogate individual laboratory data.

A steady downward trend was observed in the total percentage of samples reported as ≥1000 copies/mL. This trend was observed synchronously across platforms, and therefore could be a proxy of program success, and indicative of more-effective ART regimens, better tolerability, and subsequent improved adherence [31]. DTG, introduced as part of the first-line regimen in SA in 2019, has been shown to be more effective and well tolerated, compared to other ARVs, and most likely contributed to the decline in virological failure events, although movement from efavirenz-based regimens was initially very slow, and only completed in 2022 [32,33]. However, assay-specific differences were observed. The older m2000 assay consistently reported a lower failure rate compared to the other assays, which is consistent with other studies and is possibly due to lower sensitivity [34,35]. A slightly lower percentage of samples >1000 copies/mL was observed for the cobas assay (~1%) compared to the Alinity. 

Despite relatively consistent increases in the percentage of samples reported as <50 copies/mL initially, the trend has stagnated at about 70% since 2019, and declined in 2022. This unexpected trend, particularly the decline observed in 2022 when DTG was fully adopted, requires investigation because of individual patient management and programmatic implications. Laboratory-related factors should be excluded. Differences were observed between the Alinity and cobas assays. Although assay variation at detection of low levels of virus are well described [22,36,37], current guidelines now recommend that clinical management decisions are indicated at this threshold [7,12]. The difference between the two assays in use now does not explain the current trend but is still of concern. Direct comparisons of assay performance using clinical and analytical sample panels are underway. Furthermore, the influence of sample-collection tube types, sample integrity and sample-preparation methods on the detection of low-level viraemia should be analysed independently. 

Clinicians and program managers should be notified of assay changes and any associated influences on VL result interpretation and program metrics. Switching to improved VL assays with differences in sensitivity and detection limits may potentially affect the clinical management of individual PLWIH. Those who were previously diagnosed as suppressed or successfully treated could be diagnosed as unsuppressed or failing unchanged regimens, respectively, when their sample is tested using a different assay. Consequently, clinical management would then require more frequent follow-up visits, additional VL tests, and possibly changes to ART regimen; either empirical or guided by HIV drug resistance test results in accordance with the subscribed ART management algorithm. The 2023 South African ARV guidelines recommend a thorough assessment of elevated VL > 50 copies/mL, which includes assessment of adherence and provision of enhanced adherence counselling, and investigation into intercurrent infections and drug interactions, followed by VL test three months later [38]. Alternatively, the converse may occur, with more patients diagnosed as suppressed or successfully treated, and requiring fewer clinical visits and tests than initially anticipated. Either scenario could impact the ARV program in respect of the clinical visit and test volume projections, as well as the progress towards achieving the UNAIDS targets. 

The lessons learnt during the expansion of HIV VL testing in the SA public sector provide a blueprint for countries planning to introduce large-scale centralised laboratory testing or the scale-up of current VL testing or other high-volume molecular assays, as well as pandemic preparedness. Several advantages to centralised management of testing operations which promote better service delivery and program performance were comprehended. Firstly, a single national tender for the testing laboratories negates the need for several smaller provincial, regional, or laboratory-based tenders. Secondly, the number of tests required is much higher in a combined national tender than in smaller tenders, and the procurement thereof potentially favours lower assay prices, through economies of scale. Thirdly, the formulation of an overarching national tender implementation plan guided by the insight of the national program manager is possible. Individual laboratory needs such as staffing, shift hours and floor space availability, supplier requirements and timelines, as well the service level agreement conditions governing the award, can be cohesively integrated into the plan, providing the foundation for successful implementation. However, successful preparation and execution is contingent on effective and transparent communication among all stakeholders—the project managers, the management teams of individual laboratories and the suppliers. The outcome should be a well-considered plan that includes coordinated installation and deinstallation of instruments, pre-planned rerouting of samples, if required, management of existing reagent stock to avoid wastage, and the alignment of staff training, thereby permitting a smooth transition to testing with the new assay, limiting the disruption of service delivery, and mitigating the risk of most unforeseen events arising. The participation and cooperation of individual laboratories in planning is key to successful implementation and continuous use of the assays. The provision of standardised proficiency panels created by the VL program team assists laboratories in initiating routine testing quickly, once the instrument has been successfully installed, deemed ready for use by the supplier, and handed over to routine staff, alleviating the need to source panels themselves, which could potentially delay the start of testing. Fourthly, a monthly operations meeting per supplier, as occurs in this program, provides a convenient forum to review overall and individual laboratory instrument performance. Concerning instrument- or user-related trends such as increases in specific individual errors or run failures are identified, and interventions affected more rapidly. Furthermore, a comparison between different assays and suppliers is possible, which may be required when investigating programmatic trends at district or regional or provincial levels. Finally, the support from the program operations team is beneficial in the resolution of clinical and quality assurance queries, particularly in laboratories without specialist pathologist oversight.

An advantage of having a single laboratory service for eighty percent of the population is the use of the same LIS system in all laboratories. The interfacing of instruments to the LIS is standardised across the network, reducing implementation time and costs. Similarly, the format of result reporting is standardised across assays, facilitating easy interpretation by the clinician. Results are available electronically immediately after authorisation if accessed using an online platform. Moreover, all results are stored centrally in a corporate data warehouse, enabling easy extraction of data for reporting key performance indicators such as TAT, rejection rates and missed diagnostic opportunities. These analysed data are provided to stakeholders as reports or near-real-time dashboards at the national down to the facility level and facilitate an easier trend analysis for programmatic monitoring and evaluation. The availability of these analysed data supports directed interventions which are more likely to be beneficial to clinical management and program performance.

The VL testing program has successfully increased VL testing capacity coverage. However, there are opportunities for development. Key populations, as an example, could benefit from alternative testing strategies. Point-of-care testing is not yet in use in this program. Its introduction could enhance service provision, and investigation into the appropriate use cases should be considered.

This study had several limitations. The use of retrospective de-identified data prohibited linkage to clinical data. Viral-load-assay names for a small percentage of samples were not available on the LIS. The impact of the pre-analytical sample handling of samples on the VL result and the time between sample collection and registration could not be evaluated. Individual laboratory settings were not considered during TAT, virological failure, or suppression analysis.

## 5. Conclusions

The centralised HIV VL testing model adopted in South Africa has worked well. Successes achieved include the early provision of routine VL testing, the rapid expansion of the testing capacity, improved turnaround times and more efficient tender implementation strategies. These successes were facilitated by adopting new automated, higher-throughput VL assays, employing lessons learned from previous experiences, and the close monitoring of laboratory operations. Ongoing challenges include improving turnaround times, and increased detection of low-level viraemia, coupled with variations in assay performance near the lower limits of detection. Targeted interventions should be considered to improve turnaround times. Further evaluations are needed to identify the underlying reasons for the decline in samples of <50 copies/mL and the influence of assay performance therein.

## Figures and Tables

**Table 1:** HIV viral load test volumes for specimens received within the National Health Laboratory Service between January 2013 and December 2022. The CD4 threshold for antiretroviral treatment (ART) eligibility is indicated (cells/µL).

Year	Tested Volumes n = (%)	% Annual Change	<50 Copies/mL n = (%)	50–999 Copies/mL n = (%)	≥1000 Copies/mL n = (%)	CD4 ^&^
2013	1,961,720 (4.3)		1,011,658 (51.6)	479,547 (24.4)	470,515 (24.0)	350
2014	2,456,139 (5.4)	25.2%	1,430,823 (58.3)	487,031 (19.8)	538,285 (21.9)	500
2015	3,316,736 (7.3)	35.0%	2,129,357 (64.2)	530,720 (16.0)	656,659 (19.8)	500
2016	4,177,562 (9.2)	26.0%	2,564,106 (61.4)	807,009 (19.3)	806,447 (19.3)	All
2017	4,919,845 (10.9)	17.8%	3,083,943 (62.7)	981,249 (19.9)	854,653 (17.4)	All
2018	5,146,126 (11.4)	4.6%	3,592,546 (69.8)	785,293 (15.3)	768,287 (14.9)	All
2019	5,587,573 (12.3)	8.6%	3,849,328 (68.9)	926,400 (16.6)	811,845 (14.5)	All
2020	5,706,052 (12.6)	2.1%	4,025,777 (70.6)	918,472 (16.1)	761,803 (13.4)	All
2021	5,788,673 (12.8)	1.4%	4,046,197 (69.9)	1037,425 (17.9)	705,051 (12.2)	All
2022	6,274,438 (13.8)	8.4%	4 263 617 (68.0)	1283,954 (20.5)	726,867 (11.6)	All
Total	45,334,864 (100.0)		29,997,352 (66.2)	8,237,100 (18.2)	7,100,412 (15.7)	

^&^ The CD4 threshold for antiretroviral treatment (ART) eligibility is indicated (cells/µL).

**Table 2:** HIV viral load total turnaround time (TAT) performed within the NHLS assessed by the platform for the period between January 2013 and December 2022. The median total TAT and percentage of specimens that met the TAT target are reported.

Platform	Median TAT (in Hours)	% of Specimens that Met the TAT Target
Roche cobas 6800/8800	62	89.0%
Abbott Alinity m HIV-1	50	91.7%
Roche AmpliPrep/COBAS TaqMan	80	73.7%
Abbott RealTime HIV-1 (m2000)	77	78.2%

## Data Availability

The National Health Laboratory Service data analysed in this study are unavailable, due to patient privacy and data restrictions. Data reside in the Corporate Data Warehouse of the NHLS.
